# Meta Analysis of Gene Expression Data within and Across Species

**DOI:** 10.2174/138920208786847935

**Published:** 2008-12

**Authors:** Ana C Fierro, Filip Vandenbussche, Kristof Engelen, Yves Van de Peer, Kathleen Marchal

**Affiliations:** 1 Department of Microbial and Molecular Systems, Katholieke Universiteit Leuven, Kasteelpark Arenberg 20, 3001 Leuven, Belgium; 2 Unit Hormone Signaling and Bio-Imaging, K.L. Ledeganckstraat 35, 9000 Gent, Belgium; 3 Department of Plant Systems Biology, VIB, Technologiepark 927, 9052 Gent, Belgium

## Abstract

Since the second half of the 1990s, a large number of genome-wide analyses have been described that study gene expression at the transcript level. To this end, two major strategies have been adopted, a first one relying on hybridization techniques such as microarrays, and a second one based on sequencing techniques such as serial analysis of gene expression (SAGE), cDNA-AFLP, and analysis based on expressed sequence tags (ESTs). Despite both types of profiling experiments becoming routine techniques in many research groups, their application remains costly and laborious. As a result, the number of conditions profiled in individual studies is still relatively small and usually varies from only two to few hundreds of samples for the largest experiments. More and more, scientific journals require the deposit of these high throughput experiments in public databases upon publication. Mining the information present in these databases offers molecular biologists the possibility to view their own small-scale analysis in the light of what is already available. However, so far, the richness of the public information remains largely unexploited. Several obstacles such as the correct association between ESTs and microarray probes with the corresponding gene transcript, the incompleteness and inconsistency in the annotation of experimental conditions, and the lack of standardized experimental protocols to generate gene expression data, all impede the successful mining of these data. Here, we review the potential and difficulties of combining publicly available expression data from respectively EST analyses and microarray experiments. With examples from literature, we show how meta-analysis of expression profiling experiments can be used to study expression behavior in a single organism or between organisms, across a wide range of experimental conditions. We also provide an overview of the methods and tools that can aid molecular biologists in exploiting these public data.

## EXPRESSION PROFILING USING EST BASED META-ANALYSIS 

Expressed sequence tags (ESTs) are short segments (about 200-900 nucleotides) obtained by sequencing the 5’ and/or 3’ ends of cDNA [1]. In the release of March 2008, the public repository dbEST (http://www.ncbi.nlm.nih.gov/dbEST/) contained more than 50 million ESTs, from a wide diversity of organisms. As ESTs are obtained from diverse tissues, developmental conditions or disease stages, they unveil information on the condition-, tissue-dependent expression of a gene. ESTs are mainly useful for organisms of which the genome sequence is not yet available (and hybridization based expression profiling is not yet possible) or for higher eukaryotes in order to improve annotation. Indeed, ESTs do not only give quantitative information on a gene’s expression level, but also can provide evidence for alternative splicing and polymorphisms. The joint analysis of EST databases provides a useful resource to profile the expression of genes over different conditions. The integration of ESTs from several libraries requires, as will be outlined below, a careful selection and standardization of useful libraries.

### 1. Standardization and Data Quality

A first step towards integration across libraries is the selection of the libraries that allow for reliable EST quantification. An EST library represents a random sample of the mRNA abundance in the sampled tissue or condition, implying that the number of ESTs provides a quantification of the transcript expression level. The sequence depth of the library determines the reliability of EST derived expression quantification, in other words, the more ESTs have been sequenced from a particular library (i.e., the larger the sequence depth), the more statistically valid the derived results will be and the more rare transcripts are covered. In order to guaranty reliable quantification, either profiles are derived only for those genes for which a minimum number of ESTs are available (for example 5 or 6 ESTs) [2, 3], or the analysis only includes libraries with a minimum sequencing depth (e.g. more than 10.000 ESTs per library) [4]. Libraries for which frequencies of clones representing abundant and rare transcripts have been normalized with respect to one another, are no longer suitable for quantitative expression profiling [3]. As the absolute number of EST counts depends on the sequence depth of a library, the counts have to be standardized before they can be used as estimates of expression level, comparable between libraries [4]. 

### 2. EST Indexing

Once the right libraries have been chosen, ESTs need to be correctly ascribed to their corresponding transcripts (called indexing). EST sequences usually cover only a fraction of a transcript, and a single transcript can thus be represented by many different EST sequences. Public gene indices, such as UniGene (www.ncbi.nlm.nih.gov/UniGene/) and DFCI Gene Index (http://compbio.dfci.harvard.edu/tgi/), provide the link between available ESTs and genes [5]. Each of these public resources however introduces a different bias. By relying on different cluster strategies, Unigene rather clusters together alternative splice forms of the same gene while DFCI separates splice variants in different EST clusters. If a transcript is covered by different clusters of non overlapping ESTs, it will be represented by several clusters and there is no longer a one-to-one relationship between a transcript and a cluster of ESTs. As an alternative to these public gene indices, many research groups use their own in-house assembly of the selected ESTs [4].

### 3. Condition Annotation

For the sake of the interpretation, it is important to make sure that the tissues/cell types sampled by the libraries are clearly specified. This often requires manual curation of the library labels [5]. 

## INTEGRATING EST LIBRARIES ACROSS STUDIES (WITHIN ORGANISMS)

After standardization and indexing, EST profiling across different libraries just comes down to simple EST counting [3, 4, 6, 7]. Although still largely outnumbered by what is available for microarrays, tools exist that construct EST based expression profiles by combing data from diverse studies and laboratories. For example, DigiNorthern [8] allows extracting EST based gene expression profiles for a given gene in human and mouse respectively, while TissueInfo [9] uses ESTs to study tissue-dependent expression in the same organisms. The GBA server [10] finds co-expressed genes in respectively human, mouse and rat based on Unigene clusters. GO-Diff uses gene ontology (GO) terms to calculate functional differences between two sets of EST libraries [11]. 

EST based profiling is also often used to have a first glimpse on the gene expression prior to the sequencing of the full genome. It is mainly used to study general trends, such as the condition or tissue dependency of the gene expression. More detailed patterns such as time course experiments of a specific pathway are usually not covered. Ewing *et al.* [3] for instance, compiled a compendium of 10 rice EST libraries originating from different tissues, each of which contained about thousand ESTs. By grouping together genes with a similar expression profile over these different libraries, these authors were able to identify transcripts enriched in similar functions. More recent studies, include for instance the one of Kawaura *et al. *[4], who collected large public EST libraries to study the expression profiles of gene families in wheat and the one of Ogihara *et* *al.* [12], who used a whole series of libraries covering the wheat life cycle to detect tissue specific genes. Ronning *et al.* [2] used EST libraries of potato (*Solanum tuberosum*) to find genes associated with physiological and developmental processes such as tuber development, dormancy, and sprouting.

## INTEGRATING CROSS-SPECIES EST LIBRARIES 

When combining libraries derived from different species or organisms, the association of transcripts between different species poses an additional challenge. Ortholog association across species is not a simple task. It is well-known that, even in moderately related species, many orthologs or homologs do not show a simple one-to-one relationship. This problem of establishing a unique transcript set relation between two species is even exacerbated when also considering alternative splicing and EST assembly errors. Public databases such as HomoloGene (http://www.ncbi.nlm.nih.gov/HomoloGene/) allow the automated detection of homologs for sequenced eukaryotic genomes (see Microarray section for more details). These tools are suitable for both EST and microarray based profiling. An alternative way to link ESTs between organisms, which avoids the need for a gene-by-gene based association is to construct relationships based on functional annotations using for instance Gene Ontology [11]. 

Cross-species comparison based on EST profiles has mainly been used to identify functionally conserved homologs. As the similarity in the EST based expression profiles hints towards functional conservation, EST profile comparison between homologous genes can aid in the annotation of true orthologous relationships. Pao *et al*. [5] for instance, discovered, through comparison of EST profiles between human and mouse, that tissue-specific orthologs tend to have a more similar expression than those lacking significant tissue specificity. Orthologs for which they observed a significant disparity in expression profiles might provide an indication for neofunctionalization or subfunctionalization. It can, however, not be excluded that experimental factors, such as the heterogeneity in the tissue samples used for the library construction or the presence of an insufficient number of ESTs for these particular orthologs, contribute to the observed disparities. Fei *et al*. [6] used EST based profiling and sequence homology simultaneously to identify functionally conserved homologs (rather than orthologs) between tomato and *Arabidopsis*. The authors show that in some cases, sequence similarity alone is not sufficient to associate homologs with conserved function. 

## EXPRESSION PROFILING USING MICROARRAY BASED META-ANALYSIS

Currently, microarrays are the main technology for large-scale transcriptional gene expression profiling. By combining several independently performed microarray studies, it becomes possible to profile gene expression over a large set of conditions. Contrasting to the uniformity of the EST technology, microarrays can be manufactured in different ways, on different platforms, such as Affymetrix, Agilent, Code-link or in-house microarrays (see [13] a review). 

Each different platform requires its own optimized sample preparation, labeling, hybridization and scanning protocol, and concomitantly also a specific normalization procedure. All these differences complicate the meta-analysis of arrays performed in different research groups. Several studies have evaluated the feasibility of cross-platform and cross-laboratory integration of array experiments. While some studies show low reproducibility of expression ratios between different studies [14, 15], others present more promising results [16, 17]. Irrespective of their final conclusion, these comparative studies revealed the most important factors to be addressed when aiming at integrating data from different studies. Some of these factors are reminiscent of those described for EST profiling: 

### 1. Standarization and Data Quality

The best agreement between results of different experimental setups was reached when different labs used standardized protocols for both experimental work and data analysis [14]. Using optimized preprocessing algorithms instead of the default methods offered by the manufactures increases the comparability of the results [18]. Data obtained from microarray studies performed on the same experimental platform are usually more comparable than when different platforms are used [18]. Low data quality also seems to seriously affect reproducibility: spot quality filtering and removal of genes with low expression rates can increase the intra-platform correlation [16, 17], but results in sometimes dramatically reduced datasets. As is also the case for the selection of the right EST libraries, it is advisable to carefully test the quality of the datasets before data integration [19]. Since experiments in public databases generally have been performed independently from each other in space and time, they lack any standardization in protocols and platforms. This is the main issue which complicates their direct meta-analysis (see also below).

### 2. Probe Matching

As was also the case for combining EST profiling experiments, integrating microarray data obtained from different laboratories and/or platforms requires establishing a unique link between each gene and its corresponding probe on the different arrays. Public databases containing gene collections such as Unigene, Refseq [20] and Ensembl [21] can be used for this purpose. This linking procedure is critical and comparative studies have shown how differences in the reproducibility across platforms depend on the database used for probe matching [16, 18]. For example, as compared to probe matching using Unigene, mapping with RefSeq improved the correlation between expression ratios obtained on different platforms [16], probably because RefSeq allows a more accurate mapping of each probe to its respective splice variant than UniGene. Several probe matching tools are available for both cross-platform and cross-species applications. CROPPER [22] for example is based on the Ensembl database, while RESOURCERER [23] is based on the TIGR Gene Indices and EGO (now DFCI) and CleanEx [24] combine information from several databases, such as Unigene and RefSeq.

### 3. Condition Annotation

To allow for biological relevant data integration, it is important to select array experiments that were performed in comparable conditions. Much effort has been taken to standardize the description of the experimental protocols used for microarray experiments. The developed standard MIAME [25] defines the content required for compliant reports. It carefully describes experimental conditions, such as the genetic background of the used strains, the used media, growth conditions, triggering factors, etc., but it does not specify the format in which these data should be presented. As a result, condition annotation of a collection of microarrays obtained from public databases is still mainly a manual process where information needs to be retrieved from original publications, supplementary data and occasionally directly from the authors. After manual curation, conditions can be classified and structured to facilitate meta-analysis.

## MICROARRAY META-ANALYSIS ACROSS STUDIES FOR A SINGLE ORGANISM

**Fig. (1):**
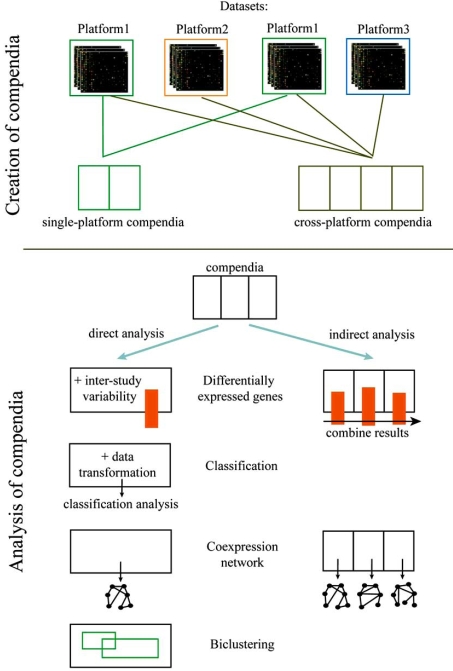
Overview of the construction and analysis of microarray compendia. Datasets generated by different laboratories can be combined to create single-platform compendia or cross-platform compendia. Methods for meta-analysis are applicable on single- and cross-platform compendia, and they can be classified as direct or indirect analyses. The methods for classification and biclustering described in this review correspond to direct analysis only.

Public databases, such as GEO [26] or ArrayExpress [27] offer a central repository of MIAME-compliant microarray data. Although these databases are an extremely rich source of information, containing thousands of experimental datasets for a particular model organism, they do not directly allow for an integrated exploration of the data between experiments (as was the case for EST experiments). An additional conversion step is needed: compendia are derived from the public resources that combine all the experiments on one particular organism (see Fig. **1**).

### Two types of compendia exist

1. Single-platform compendia combine all data on a particular organism that were obtained from one specific platform. Focusing on a single platform makes both the between-experiment normalization and the probe-matching relatively straightforward. Normalization is performed with the uniform platform-specific normalization procedure. Most single-platform compendia focus on Affymetrix as it turned out to be one of the more robust and reproducible platforms [14, 18]. Examples are, for instance, Genevestigator [28], initially developed for *Arabidopsis,* but now being extended to other species such as human and mouse, and M3D [29], which offers Affy-based compendia for three microbial organisms (*E. coli*, yeast and *Shewanella oneidensis*). Such single platform compendia are more straightforward to use for direct meta-analysis (see below).
2. Cross-platform compendia include data from different platforms and often combine data from both one- and two-channel microarrays. These compendia are topic-specific, collecting all the publicly available experimental information related to the topic of interest. ITTACA [30] and ONCOMINE [31], for instance, focus on cancer in human, GAN [19] on aging in several species. They collect already normalized datasets (ITTACA and GAN) or apply a simple scaling normalization method (ONCOMINE). In the examples mentioned above, Unigene is used for probe matching. Because of the heterogeneity in platforms, usually each experimental set is analyzed separately and independent analyses are subsequently combined or compared across datasets (indirect meta-analysis/see below).

Standard microarray analysis such as detecting differentially expressed genes, clustering gene expression profiles, classification or reconstructing gene co-expression networks are also applicable on large expression compendia. However, the inter-study variability caused by the use of different platforms and/or experimental procedures complicates the analysis. The fact that compendia contain a plethora of different conditions makes the interpretation and analysis also less straightforward than is the case for the analysis of a single experiment. In the following, we describe standard microarray analysis protocols that have been specifically adapted towards their use on a compilation of different independent datasets, i.e., towards meta-analysis. From a statistical point of view, such meta-analysis is interesting as for single experiments the number of replicated conditions is usually small. By combining results from different studies that address a set of related research hypotheses, the number of replicates and the power of the statistical tests will increase [32]. 

For the meta-analysis of microarrays, direct and indirect methods have been developed (Fig. **1**), which can be applied on both, single and cross-platform compendia:

- For direct meta-analysis, microarray analysis procedures (such as clustering, network reconstruction) are applied to the compendium as a whole. Consistent sources of variation related to differences in experimental set up have to be removed prior to these subsequent analyses.
- The indirect analysis first applies the desired microarray analysis procedure on each single data set within the compendium separately and subsequently combines the derived results.

#### Detection of Differentially Expressed Genes

Many independently performed experiments exist in which a similar process is studied in comparable conditions, for example for the profiling of gene expression in a specific cancer type. Such datasets are ideally suited for the indirect meta-analysis of genes that are differentially expressed between two biological conditions. Rhodes *et al.* [33] combined four independent datasets to identify genes dysregulated in prostate cancer. For each gene in each dataset a p-value was obtained as an indication of the probability that the gene was differentially expressed. P-values for the different datasets were subsequently aggregated to provide an overall estimate of the gene’s significance of being differentially expressed during prostate cancer. This indirect approach aims at validating and statistically assessing the results across datasets. However, it is still limited in its use as it requires that the different datasets which are combined test the exact same conditions. To extend this approach to a compendium of more heterogeneous conditions, Rhodes *et al.* [34] first subdivided the compendium in subsets according to predefined comparisons of interest (e.g., cancer versus normal, undifferentiated versus well differentiated cancer). By searching for subsets of genes that are frequently differentially expressed in a subset, but not necessarily in all the conditions within the subset they introduced more flexibility towards the heterogeneity of the data.

Direct approaches that first standardize raw expression values by removing the inter-study variability and subsequently use these standardized data for detecting differentially expressed genes, have also been applied. Such direct meta-analysis becomes useful if the number of studies included in the analysis is sufficiently large to reliably estimate the inter-study variability [35]. Although they can enhance the power for detecting differentially expressed genes, these methods are still rarely used. An example is given by Choi *et al.* [36] who use a statistical model to estimate from a standardized mean difference in gene expression between two conditions the differential expression. The statistical model they use takes into account both the within-study (different replicas) and between-study variability. Hu *et al.* [37] have extended the model proposed by Choi *et al.* [36] with quality measures derived from of the original raw data. Stevens *et* *al*. [38] proposed an alternative for the standardized mean difference [36] as estimator for differential expression specific for Affymetrix data.

Linear models such as LIMMA [39] and ANOVA [40] were originally developed to search for differentially expressed genes or to reconstruct profiles from complex microarray designs derived from a single experiment [41]. By explicitly including in these models, a factor that compensates for consistent sources of variation across different experiments, these techniques can be adapted in a straightforward way for direct cross-experiment analysis. Park *et al.* [42] propose an ANOVA model accounting for the inter-study variability while Gilks *et al.* [43] propose a multiple regression model to combine different expression values profiles under the similar experimental conditions. In general, direct and indirect approaches give different results. When taking the results obtained by the analysis of a single experiments as a reference, direct meta-analysis of multiple experiments detects more differentially expressed genes while indirect analysis tend to result in a more restricted gene list which corresponds grosso modo to the intersection of the sets of differentially expressed genes obtained by each of the single experiments. 

#### Classification Techniques 

Also supervised classification techniques benefit from the larger number of samples in a microarray compendium. Their aim is to find genes or features (combinations of genes) that can discriminate two classes, such as normal and cancer samples, or between phenotypes. Some direct analysis strategies for classification convert the expression values into sorted gene lists, and afterwards use the relative rank (the gene position in the sorted list) within each condition for further analysis. Although this rank-based transformation results in the loss of the absolute values of gene expression, it guarantees comparability between the different experiments within a compendium while still providing sufficient information for classification [44, 45]. Several studies showed that classifiers trained with a compendium outperform classifiers based on a single dataset [44, 45].

#### Comparisons Across Platforms

Besides for classification purposes, also methods have been developed that allow making general comparisons between different datasets. Prior to the comparison, the complexity of a compendium is reduced by defining linear combinations of genes that describe the main biological aspects contained within the compendium (metagenes) [46]. A reference (model set) compendium is used to define these metagenes. Data from other platforms (test sets) can subsequently be compared with the reference set by projecting the test sets onto the meta-genes of the reference. 

The previously described comparison method is gene based and as such the analysis is restricted to only those genes which are in common between all the arrays of the used compendium. In some cases this results in the exclusion of thousands of genes from the analysis. By using a condition-based approach, Culhane *et al*. [47] circumvented this problem. These authors developed “Co-inertia analysis” (CIA), an approach that identifies common trends or co-relationships between the conditions of two different datasets. CIA is accomplished by finding successive orthogonal axes from the two datasets with maximum squared covariance using correspondence analysis. By applying their method to cancer related datasets, they were able to distinguish between cancer cell types and concomitantly identified the genes of which the expression contributed to the observed global expression differences between the cell types. 

#### Reconstruction of Co-Expression Networks

Genes that are coexpressed over a certain number of conditions suggest that these genes might be functionally related or even co-regulated. From a large-enough number of samples in a compendium, a coexpression network can be inferred [48-52]. A coexpression network is a graph-based representation of pairwisely coexpressed genes. A node represents a gene and an edge indicates that the connected genes are coexpressed in the network. The pairwise co-expression is usually assessed by Pearson correlation [48, 50] or mutual information [51]. In general, the significance of each edge is calculated by assigning a “relevance score”, which is based on rank scores [48, 50, 51] or statistical tests [49, 52] to select the most significant interactions which define the network. These networks are then further subdivided into highly connected subgraphs which correspond to modules of functionally related genes [48-50, 52]. 

As coexpression networks are by definition condition-dependent, not all interactions are valid in all conditions. To cope with this condition dependency, heterogeneous compendia are subdivided before calculating the coexpression networks. In an indirect approach, the compendium is subdivided into subsets according to the different experiments (datasets) from which its is composed [49, 52]. In a direct approach, the compendium is analyzed as a whole [51] or divided according to predefined categories, such as different tissues. In the latter case a predefined category does not necessarily correspond to a single experiment as is the case with an indirect analysis but can be composed of different experiment sets profiling similar conditions [48, 50]. Choi *et al*. [48] used a direct approach of coexpression network inference to search for differences in expression between cancer or normal tissues by comparing coexpression networks extracted from compendia containing expression data from the respective tissues. In some studies, a reference coexpression network is derived from a first compendium and compared with an independent compendium to infer the subnetworks that are affected by measuring the effects of a specific treatment [50]. Coexpression networks have also been used to refine gene annotation by studying the condition-dependency of a particular interaction in the coexpression network [49]. 

#### (Bi)Clustering and Gene Modules Inference

Another common task in microarray analysis is the clustering of genes that share a similar gene expression pattern across the tested conditions. Standard clustering methods are successful in grouping together co-expressed genes in relatively small datasets or larger datasets that focus on a particular condition. However, searching for patterns of co-expression that extend over all conditions in a compendium that is heterogeneous with respect to these conditions, is little useful. In general we can expect that most genes are only affected by a small subset of these conditions. Moreover, genes may participate in different pathways and thus could be part of several overlapping clusters, a problem that is also not tackled by standard clustering approaches. To analyze large compendia, module detection or bicluster approaches are therefore more appropriate. These algorithms not only select genes which are co-expressed but also the conditions these genes are co-expressed in [53, 54]. 

Query based approaches allow searching compendia for genes that are coexpressed with a certain gene of interest, e.g., a potential drug target. These query driven methods report the set of genes with similar behavior to the query genes and the conditions under which these genes are coexpressed [55, 56]. To deal with the condition-dependency of the co-expression, Hibbs *et al*. [56] decompose the compendium into its original experiment sets and assess coexpression in each of the individual sets. Dhollander *et al*. [55] do not use any prior condition partitioning of the compendium but rely on a bicluster strategy for deriving, simultaneously with the coexpressed genes, the conditions under which these genes are coexpressed. Since the query genes can be involved in several pathways and functions, Dhollander *et al*. [55] apply a range of different parameter settings to detect small biclusters with homogeneous co-expression profiles as well as bigger biclusters with more heterogeneous profiles.

## DATA INTEGRATION ACROSS SPECIES 

With all these microarray platforms being set up for several model organisms, the comparison of expression profiles across species offers new opportunities towards studying network and pathway evolution. Cross-species analyses exist which compare closely related species, such as different subspecies of yeast or *Drosophila* (*Drosophila melanogaster*) [57, 58], or more evolutionary distant organisms, such as human (*Homo sapiens*), fly, yeast (*Saccharomyces cerevisiae*) and *Caenorhabditis elegans *[59]. Comparative studies either focus on a particular biological process, such as aging [60] or metamorphosis [57], or on more global comparisons , to study for example, core biological functions such as the cell cycle, secretion, and protein expression [59]. Because compendia are usually heterogeneous in the conditions they assess for the different organisms, specifically designed datasets profiling comparable conditions for the different organisms are more suitable for cross-species analysis.

As was also the case for cross-species analysis using EST profiling, most microarray based cross-species analyses rely on the mapping of orthologous genes between the different organisms. To this end, similar tools as described for EST analysis can be used (CROPPER and RESOURCERER). Ortholog identification usually relies on sequence similarity using bidirectional best hits (BBH) [59] or sequence similarity combined with phylogenetic analysis [60]. However, due to evolutionary phenomena such as sub- and neofunctionalization, associations based on sequence similarity do not always imply similar functionalities. Therefore, instead of identifying orthologs prior to the cross-species expression analysis, one can use the expression data besides the sequence similarity to simultaneously search for sequence and functional conservation. Bergmann *et al.* [61], for instance, developed to this end a two-step approach in which they first, starting from a group of coexpressed genes in one organism, identified the corresponding homologs in a second organism. In a second step only homologs that also appeared coexpressed in the second reference organism are retained as functional homologs. Lefebvre *et al*. [62] defined a single measure to detect functionally conserved genesets between *C. elegans* and *Drosophila* that includes simultaneously the sequence alignment score between homologous genes and a within species gene coexpression score [62]. As such, expression data can help refining the ortholog identification [61, 62]. 

Because of the heterogeneity in platforms and compendia, the meta-analysis of different datasets across organisms is much less straightforward than with ESTs and as a consequence, no standardized procedures exist. Studies which use a homogeneous compendia, i.e. the dataset for both organisms contain similar conditions, rely on differences of gene expression to compare the changes in the transcriptional response between organisms [57]. The correlation between the log ratios of all genes is used as a global indication of how much the conditions are comparable between the different organisms [60]. Rifkin *et al*. [57] for example studied “evolutionary variation” of gene expression in *Drosophila *at the onset of metamorphosis by comparing to what extent orthologous genes exhibiting developmental changes during metamorphosis in one species were no longer differentially expressed during the same process in other members of the species. McCaroll *et al*. [60] compared gene expression ratios to assess the similarity of the process of aging between *C. elegans* and *D. melanogaster*. To compare networks between species, the concept of coexpression networks has also been applied (see above). Lelandais *et al.* [63], for instance, compared the sporulation network between budding and fission yeasts using for both organisms similarly designed time series experiments. The authors proposed a method that superimposes the two species specific coexpression networks by taking into account the structure of each individual network and the orthologous relations between the species.

When the compendia for each organism tend to be more heterogeneous in conditions, individual gene profiles are no longer comparable across organisms, but the mutual relation between genes, can still be compared between species [58, 59, 61]. Stuart *et al*. [59] for instance used coexpression networks to compare expression networks in *H. sapiens*, *D. melanogaster*, *S. cerevisiae* and *C. elegans.* The authors started from a set of genes that exhibit sequence conservation in the different species studies. Subsequently, they identified the coexpression network of those genes for which the representatives are consistently coexpressed in all species studied. This conservation of genes being coexpressed over different species is an indication of conservation of coexpression throughout evolution. Also based on the conservation of coexpression, Ihmels *et al*. [58] developed the Differential Clustering Algorithm (DCA) to capture differences in expression patterns between two yeast species *C. albicans* and *S. cerevisiae*. The algorithm is used to determine if the expression of a group of coexpressed genes in one organism is fully, partially, or not at all conserved in the other organism. To facilitate the analysis and interpretation of the results, the authors focused their analysis on gene sets which are predefined by sharing common regulatory motifs or belonging to the same GO categories. They discovered that most of the differences in expression modularity occurred in genes involved in mitochondrial processes. In contrast to previous studies which calculated the coexpression between genes over all the conditions, Bergmann *et al*. take into account the condition-dependency of the coexpression by relying on a biclustering approach (see before) [61]. They compared global expression patterns between *S. cerevisiae*, *C. elegans*, *E. coli*, *A. thaliana*, *D. melanogaster*, and *H. sapiens*. The iterative signature algorithm (ISA) [64] was applied to decompose the compendium of each organism in co-expressed modules. Next, they compared to what extent each of these organisms shared homologous modules, i.e. a module of coexpressed genes in the reference species (yeast in their study) of which the orthologs or homologs are also coexpressed in the other species. The difficulty with biclustering is that the concept of a biological module, being a set of coexpressed genes and the conditions under which they are coexpressed is hard to formalize mathematically. Depending on the ISA parameter resolution, a gene can belong to a whole series of overlapping modules. At low resolution ISA finds few large loosely coexpressed modules, while at a high resolution ISA finds smaller but more tightly coexpressed modules. This complicates comparing modules over different species because a module is not uniquely defined. Bergmann *et al*. [61] tackle this issue by introducing high-order regulatory structures or module trees that show the relation between the modules obtained at different resolutions and comparing these module trees across the species instead of the single modules. 

## COMPARISON BETWEEN ESTS AND MICROARRAY BASED PROFILING

Although both ESTs and microarrays are used to measure gene expression and theoretically describe the same process of transcriptional regulation, both methods for expression profiling have largely been developed independently from each other. The question remains as to what extent both techniques agree with each other in describing similar transcriptional processes. 

The current sensitivity of microarrays is probably still insufficient to detect relevant changes in expression for low abundance genes such as transcription factors [65]. For EST based profiling, in principle, good estimates of gene expression, even for lowly expressed genes can be obtained provided a sufficient number of ESTs is available (sequence depth is sufficient and the library is large). However, this is usually not the case for classical sequence based profiling techniques because of the required cost and effort to generate such libraries. Another problem shared by the EST- and microarray based profiling techniques is their specificity in assigning each probe or sequence to a unique transcript or gene. Microarray probes that are too short or ill-designed will lead to cross-hybridization, a problem which is exacerbated for orthologs and paralogs belonging to the same protein family [66]. For EST based profiling, making the distinction between closely related members of a gene family would in theory be less of a problem because accurate EST sequencing results in discriminating nucleotide polymorphisms for each of the sequences [67]. However, such accuracy is usually not yet obtained with the classical EST based profiling techniques. For ESTs there are also other reasons why the mapping between a transcript and a gene is not always unique. For instance, when non overlapping ESTs are derived from the 5’ and 3’ extreme ends of a long transcript, their reads will erroneously be assigned to different genes. Also EST libraries are often incomplete because small transcripts are removed during library construction, and sequences which are difficult to clone or which lead to instabilities in the vector are often missed. 

Comparative studies focusing on the detection of differentially expressed genes among tissues showed clear differences between results obtained by EST versus microarray profiling [5, 67, 68]. In general, at this stage the use of sequence based profiling techniques is probably less suitable for quantitative expression analysis than array based expression profiling, mainly because of incomplete and insufficiently large libraries and sequence coverage. However, with the use of the novel sequence strategies such as reversible terminator sequencing [69] or pyrosequencing technology [70], this situation can be quickly reversed. Massive parallel pyrosequencing strategies allow for the direct sequencing of cDNA, obviate the need for a library construction, and can obtain a much higher coverage at a lower cost and time. They thus overcome most of the limitations of the classical EST based profiling techniques [71, 72]. 

## CONCLUSION

The combination of relatively small-scaled publicly available profiling experiments increases the power of statistical tests and improves the detection of interesting genes by identifying subtle signals that seem recurrent across multiple experiments. Moreover, by generating a compendium of experiments, a much wider range of conditions is covered for a particular organism. This not only allows increasing the scope of the own small-scale study, but also contributes to the understanding of the organism at a more global level. Integrated analysis of experiments across species improves functional annotation and true ortholog identification and will eventually lead to the basic understanding of how expression networks evolved. Meta-analysis of gene expression data thus holds much promise. Microarrays are already customarily used and in principle very large compendia for model organisms can already be compiled. With the adoption of the many novel ultra fast sequencing technologies, the sequence based expression profiling will definitely see a revival.

With the increasing number of high throughput technologies, we can expect that compendia for other “omics data” will also grow at an increasing pace. Each compendium provides a snap shot of the condition-dependent changes at a certain cellular level. A huge challenge remains of how a comprehensive view of the cellular machinery can be built by combining all these individual snap shots [73-75]. An important and often overlooked issue with the meta-analysis of biological data is the context-dependency, the condition dependency of the interactions, their timing, and their location. Most of the representations of a network obtained so far are static. Taking into account context will require the development of appropriate analysis techniques, such as for instance biclustering (see higher), but more importantly, a more formalized and standardized way of describing experimental context.

## References

[1] Adams MD, Kelley JM, Gocayne JD, Dubnick M, Poly-meropoulos MH, Xiao H, Merril CR, Wu A, Olde B, Moreno RF, Kerlavage AR, McCombie WR, Venter JC (1991). Complementary DNA sequencing: expressed sequence tags and human genome project. Science.

[2] Ronning CM, Stegalkina SS, Ascenzi RA, Bougri O, Hart AL, Utterbach TR, Vanaken SE, Riedmuller SB, White JA, Cho J, Pertea GM, Lee Y, Karamycheva S, Sultana R, Tsai J, Quackenbush J, Griffiths HM, Restrepo S, Smart CD, Fry WE, Van Der Hoeven R, Tanksley S, Zhang P, Jin H, Yamamoto ML, Baker BJ, Buell CR (2003). Comparative analyses of potato expressed sequence tag libraries. Plant Physiol.

[3] Ewing RM, Ben Kahla A, Poirot O, Lopez F, Audic S, Claverie JM (1999). Large-scale statistical analyses of rice ESTs reveal correlated patterns of gene expression. Genome Res.

[4] Kawaura K, Mochida K, Ogihara Y (2005). Expression profile of two storage-protein gene families in hexaploid wheat revealed by large-scale analysis of expressed sequence tags. Plant Physiol.

[5] Pao SY, Lin WL, Hwang MJ (2006). In silico identification and comparative analysis of differentially expressed genes in human and mouse tissues. BMC Genomics.

[6] Fei Z, Tang X, Alba RM, White JA, Ronning CM, Martin GB, Tanksley SD, Giovannoni JJ (2004). Comprehensive EST analysis of tomato and comparative genomics of fruit ripening. Plant J.

[7] Fierro AC, Thuret R, Coen L, Perron M, Demeneix BA, Wegnez M, Gyapay G, Weissenbach J, Wincker P, Ma-zabraud A, Pollet N (2007). Exploring nervous system transcriptomes during embryogenesis and metamorphosis in Xenopus tropicalis using EST analysis. BMC Genomics.

[8] Wang J, Liang P (2003). DigiNorthern, digital expression analysis of query genes based on ESTs. Bioinformatics.

[9] Skrabanek L, Campagne F (2001). TissueInfo: high-throughput identification of tissue expression profiles and specificity. Nucleic Acids Res.

[10] Wu X, Walker MG, Luo J, Wei L (2005). GBA server: EST-based digital gene expression profiling. Nucleic Acids Res.

[11] Chen Z, Wang W, Ling X B, Liu JJ, Chen L (2006). GO-Diff: mining functional differentiation between EST-based transcriptomes. BMC Bioinformatics.

[12] Ogihara Y, Mochida K, Nemoto Y, Murai K, Yamazaki Y, Shin IT, Kohara Y (2003). Correlated clustering and virtual display of gene expression patterns in the wheat life cycle by large-scale statistical analyses of expressed sequence tags. Plant J.

[13] Sasik R, Woelk CH, Corbeil J (2004). Microarray truths and consequences. J Mol. Endocrinol.

[14] Bammler T, Beyer RP, Bhattacharya S, Boorman GA, Boyles A, Bradford BU, Bumgarner RE, Bushel PR, Chaturvedi K, Choi D, Cunningham ML, Deng S, Dressman HK, Fannin RD, Farin FM, Freedman JH, Fry RC, Harper A, Humble MC, Hurban P, Kavanagh TJ, Kaufmann WK, Kerr KF, Jing L, Lapidus JA, Lasarev MR, Li J, Li Y J, Lobenhofer EK, Lu X, Malek RL, Milton S, Nagalla SR, O'Malley J P, Palmer VS, Pattee P, Paules RS, Perou CM, Phillips K, Qin LX, Qiu Y, Quigley SD, Rodland M, Rusyn I, Samson LD, Schwartz DA, Shi Y, Shin JL, Sieber SO, Slifer S, Speer MC, Spencer PS, Sproles DI, Swenberg JA, Suk WA, Sullivan RC, Tian R, Tennant RW, Todd SA, Tucker CJ, Van Houten B, Weis BK, Xuan S, Zarbl H (2005). Standardizing global gene expression analysis between laboratories and across platforms. Nat. Methods.

[15] Tan PK, Downey TJ, Spitznagel EL Jr, Xu P, Fu D, Dimitrov DS, Lempicki RA, Raaka BM, Cam MC (2003). Evaluation of gene expression measurements from commercial microarray platforms. Nucleic Acids Res.

[16] Kuo WP, Liu F, Trimarchi J, Punzo C, Lombardi M, Sarang J, Whipple ME, Maysuria M, Serikawa K, Lee SY, McCrann D, Kang J, Shearstone JR, Burke J, Park DJ, Wang X, Rector TL, Ricciardi-Castagnoli P, Perrin S, Choi S, Bumgarner R, Kim JH, Short GF 3rd, Freeman MW, Seed B, Jensen R, Church GM, Hovig E, Cepko CL, Park P, Ohno-Machado L, Jenssen TK (2006). A sequence-oriented comparison of gene expression measurements across different hybridization-based technologies. Nat. Biotechnol.

[17] Shi L, Tong W, Fang H, Scherf U, Han J, Puri R K, Frueh FW, Goodsaid FM, Guo L, Su Z, Han T, Fuscoe JC, Xu ZA, Patterson TA, Hong H, Xie Q, Perkins RG, Chen JJ, Casciano DA (2005). Cross-platform comparability of microarray technology: intra-platform consistency and appropriate data analysis procedures are essential. BMC Bioinformatics.

[18] Irizarry RA, Warren D, Spencer F, Kim IF, Biswal S, Frank BC, Gabrielson E, Garcia JG, Geoghegan J, Germino G, Griffin C, Hilmer SC, Hoffman E, Jedlicka AE, Kawasaki E, Martinez-Murillo F, Morsberger L, Lee H, Petersen D, Quackenbush J, Scott A, Wilson M, Yang Y, Ye SQ, Yu W (2005). Multiple-laboratory comparison of microarray platforms. Nat. Methods.

[19] Pan F, Chiu CH, Pulapura S, Mehan MR, Nunez-Iglesias J, Zhang K, Kamath K, Waterman MS, Finch CE, Zhou XJ (2007). Gene Aging Nexus: a web database and data mining platform for microarray data on aging. Nucleic Acids Res.

[20] Pruitt KD, Tatusova T, Maglott DR (2007). NCBI reference sequences (RefSeq): a curated non-redundant sequence database of genomes, transcripts and proteins. Nucleic Acids Res.

[21] Hubbard TJ, Aken BL, Beal K, Ballester B, Caccamo M, Chen Y, Clarke L, Coates G, Cunningham F, Cutts T, Down T, Dyer SC, Fitzgerald S, Fernandez-Banet J, Graf S, Haider S, Hammond M, Herrero J, Holland R, Howe K, Howe K, Johnson N, Kahari A, Keefe D, Kokocinski F, Kulesha E, Lawson D, Longden I, Melsopp C, Megy K, Meidl P, Ouverdin B, Parker A, Prlic A, Rice S, Rios D, Schuster M, Sealy I, Severin J, Slater G, Smedley D, Spudich G, Trevanion S, Vilella A, Vogel J, White S, Wood M, Cox T, Curwen V, Durbin R, Fernandez-Suarez XM, Flicek P, Kasprzyk A, Proctor G, Searle S, Smith J, Ureta-Vidal A, Birney E (2007). Ensembl 2007. Nucleic Acids Res.

[22] Paananen J, Storvik M, Wong G (2006). CROPPER: a metagene creator resource for cross-platform and cross-species compendium studies. BMC Bioinformatics.

[23] Tsai J, Sultana R, Lee Y, Pertea G, Karamycheva S, An-tonescu V, Cho J, Parvizi B, Cheung F, Quackenbush J (2001). RESOURCERER: a database for annotating and linking microarray resources within and across species. Genome Biol.

[24] Praz V, Jagannathan V, Bucher P (2004). CleanEx a database of heterogeneous gene expression data based on a consistent gene nomenclature. Nucleic Acids Res.

[25] Brazma A, Hingamp P, Quackenbush J, Sherlock G, Spell-man P, Stoeckert C, Aach J, Ansorge W, Ball CA, Causton HC, Gaasterland T, Glenisson P, Holstege FC, Kim IF, Markowitz V, Matese JC, Parkinson H, Robinson A, Sarkans U, Schulze-Kremer S, Stewart J, Taylor R, Vilo J, Vingron M (2001). Minimum information about a microarray experiment (MIAME)-toward standards for microarray data. Nat. Genet.

[26] Barrett T, Troup DB, Wilhite SE, Ledoux P, Rudnev D, Evangelista C, Kim IF, Soboleva A, Tomashevsky M, Edgar R (2007). NCBI GEO: mining tens of millions of expression profiles--database and tools update. Nucleic Acids Res.

[27] Parkinson H, Kapushesky M, Shojatalab M, Abeygunawardena N, Coulson R, Farne A, Holloway E, Kolesnykov N, Lilja P, Lukk M, Mani R, Rayner T, Sharma A, William E, Sarkans U, Brazma A (2007). ArrayExpress--a public database of microarray experiments and gene expression profiles. Nucleic Acids Res.

[28] Laule O, Hirsch-Hoffmann M, Hruz T, Gruissem W, Zimmermann P (2006). Web-based analysis of the mouse transcriptome using Genevestigator. BMC Bioinformatics.

[29] Faith JJ, Driscoll ME, Fusaro VA, Cosgrove EJ, Hayete B, Juhn FS, Schneider SJ, Gardner TS (2008). Many Microbe Microarrays Database: uniformly normalized Affymetrix compendia with structured experimental metadata. Nucleic Acids Res.

[30] Elfilali A, Lair S, Verbeke C, La Rosa P, Radvanyi F, Baril-lot E (2006). ITTACA: a new database for integrated tumor transcriptome array and clinical data analysis. Nucleic Acids Res.

[31] Rhodes DR, Kalyana-Sundaram S, Mahavisno V, Varambally R, Yu J, Briggs BB, Barrette TR, Anstet MJ, Kincead-Beal C, Kulkarni P, Varambally S, Ghosh D, Chinnaiyan AM (2007). Oncomine 3.0: genes, pathways, and networks in a collection of 18,000 cancer gene expression profiles. Neoplasia.

[32] Sutton AJ, Abrams KR, Jones DR (2001). An illustrated guide to the methods of meta-analysis. J. Eval. Clin. Pract.

[33] Rhodes DR, Barrette TR, Rubin MA, Ghosh D, Chinnaiyan AM (2002). Meta-analysis of microarrays: interstudy validation of gene expression profiles reveals pathway dysregulation in prostate cancer. Cancer Res.

[34] Rhodes DR, Yu J, Shanker K, Deshpande N, Varambally R, Ghosh D, Barrette T, Pandey A, Chinnaiyan AM (2004). Large-scale meta-analysis of cancer microarray data identifies common transcriptional profiles of neoplastic transformation and progression. Proc. Natl. Acad. Sci. USA.

[35] Conlon EM, Song JJ, Liu A (2007). Bayesian meta-analysis models for microarray data: a comparative study. BMC Bioinformatics.

[36] Choi JK, Yu U, Kim S, Yoo OJ (2003). Combining multiple microarray studies and modeling interstudy variation. Bioinformatics.

[37] Hu P, Greenwood CM, Beyene J (2005). Integrative analysis of multiple gene expression profiles with quality-adjusted effect size models. BMC Bioinformatics.

[38] Stevens JR, Doerge RW (2005). Combining Affymetrix microarray results. BMC Bioinformatics.

[39] Smyth GK (2004). Linear models and empirical bayes methods for assessing differential expression in microarray experiments. Stat. Appl. Genet. Mol. Biol.

[40] Kerr MK, Martin M, Churchill GA (2000). Analysis of variance for gene expression microarray data. J. Comput. Biol.

[41] Fierro AC, Thuret R, Engelen K, Bernot G, Marchal K, Pollet N (2008). Evaluation of time profile reconstruction from complex two-color microarray designs. BMC Bioinformatics.

[42] Park T, Yi SG, Shin YK, Lee S (2006). Combining multiple microarrays in the presence of controlling variables. Bioinformatics.

[43] Gilks WR, Tom BD, Brazma A (2005). Fusing microarray experiments with multivariate regression. Bioinformatics.

[44] Warnat P, Eils R, Brors B (2005). Cross-platform analysis of cancer microarray data improves gene expression based classification of phenotypes. BMC Bioinformatics.

[45] Xu L, Tan AC, Naiman DQ, Geman D, Winslow RL (2005). Robust prostate cancer marker genes emerge from direct integration of inter-study microarray data. Bioinformatics.

[46] Tamayo P, Scanfeld D, Ebert BL, Gillette MA, Roberts CW, Mesirov JP (2007). Metagene projection for cross-platform, cross-species characterization of global transcriptional states. Proc. Natl. Acad. Sci. USA.

[47] Culhane AC, Perriere G, Higgins DG (2003). Cross-platform comparison and visualisation of gene expression data using co-inertia analysis. BMC Bioinformatics.

[48] Choi JK, Yu U, Yoo OJ, Kim S (2005). Differential coexpression analysis using microarray data and its application to human cancer. Bioinformatics.

[49] Huang Y, Li H, Hu H, Yan X, Waterman MS, Huang H, Zhou XJ (2007). Systematic discovery of functional modules and context-specific functional annotation of human genome. Bioinformatics.

[50] Ucar D, Neuhaus I, Ross-MacDonald P, Tilford C, Parthasarathy S, Siemers N, Ji RR (2007). Construction of a reference gene association network from multiple profiling data: application to data analysis. Bioinformatics.

[51] Faith JJ, Hayete B, Thaden JT, Mogno I, Wierzbowski J, Cottarel G, Kasif S, Collins JJ, Gardner TS (2007). Large-scale mapping and validation of Escherichia coli transcriptional regulation from a compendium of expression profiles. PLoS Biol.

[52] Yan X, Mehan MR, Huang Y, Waterman MS, Yu PS, Zhou XJ (2007). A graph-based approach to systematically reconstruct human transcriptional regulatory modules. Bioinformatics.

[53] Van den Bulcke T, Lemmens K, Van de Peer Y, Marchal K (2006). Inferring Transcriptional Networks by Mining Omics Data. Current Bioinformatics.

[54] Lemmens K, Dhollander T, De Bie T, Monsieurs P, Engelen K, Smets B, Winderickx J, De Moor B, Marchal K (2006). Inferring transcriptional modules from ChIP-chip, motif and microarray data. Genome Biol.

[55] Dhollander T, Sheng Q, Lemmens K, De Moor B, Marchal K, Moreau Y (2007). Query-driven module discovery in microarray data. Bioinformatics.

[56] Hibbs MA, Hess DC, Myers CL, Huttenhower C, Li K, Troyanskaya OG (2007). Exploring the functional landscape of gene expression: directed search of large microarray compendia. Bioinformatics.

[57] Rifkin SA, Kim J, White KP (2003). Evolution of gene expression in the Drosophila melanogaster subgroup. Nat. Genet.

[58] Ihmels J, Bergmann S, Berman J, Barkai N (2005). Comparative gene expression analysis by differential clustering approach: application to the Candida albicans transcription program. PLoS Genet.

[59] Stuart JM, Segal E, Koller D, Kim SK (2003). A gene-coexpression network for global discovery of conserved genetic modules. Science.

[60] McCarroll SA, Murphy CT, Zou S, Pletcher SD, Chin CS, Jan YN, Kenyon C, Bargmann CI, Li H (2004). Comparing genomic expression patterns across species identifies shared transcriptional profile in aging. Nat. Genet.

[61] Bergmann S, Ihmels J, Barkai N (2004). Similarities and differences in genome-wide expression data of six organisms. PLoS Biol.

[62] Lefebvre C, Aude JC, Glemet E, Neri C (2005). Balancing protein similarity and gene co-expression reveals new links between genetic conservation and developmental diversity in invertebrates. Bioinformatics.

[63] Lelandais G, Vincens P, Badel-Chagnon A, Vialette S, Jacq C, Hazout S (2006). Comparing gene expression networks in a multidimensional space to extract similarities and differences between organisms. Bioinformatics.

[64] Bergmann S, Ihmels J, Barkai N (2003). Iterative signature algorithm for the analysis of large-scale gene expression data. Phys. Rev. E Stat. Nonlin. Soft Matter Phys.

[65] Draghici S, Khatri P, Eklund AC, Szallasi Z (2006). Reliability and reproducibility issues in DNA microarray measurements. Trends Genet.

[66] Casneuf T, Van de Peer Y, Huber W (2007). In situ analysis of cross-hybridisation on microarrays and the inference of expression correlation. BMC Bioinformatics.

[67] Fernandes J, Brendel V, Gai X, Lal S, Chandler VL, Elumalai RP, Galbraith DW, Pierson EA, Walbot V (2002). Comparison of RNA expression profiles based on maize expressed sequence tag frequency analysis and micro-array hybridization. Plant Physiol.

[68] Huminiecki L, Lloyd AT, Wolfe KH (2003). Congruence of tissue expression profiles from Gene Expression Atlas, SAGEmap and TissueInfo databases. BMC Genomics.

[69] Bennett ST, Barnes C, Cox A, Davies L, Brown C (2005). Toward the 1,000 dollars human genome. Pharmacogenomics.

[70] Margulies M, Egholm M, Altman WE, Attiya S, Bader JS, Bemben LA, Berka J, Braverman MS, Chen YJ, Chen Z, Dewell SB, Du L, Fierro JM, Gomes XV, Godwin BC, He W, Helgesen S, Ho CH, Irzyk GP, Jando SC, Alenquer ML, Jarvie TP, Jirage KB, Kim JB, Knight JR, Lanza JR, Leamon JH, Lefkowitz SM, Lei M, Li J, Lohman KL, Lu H, Makhijani VB, McDade KE, McKenna MP, Myers EW, Nickerson E, Nobile JR, Plant R, Puc BP, Ronan MT, Roth GT, Sarkis GJ, Simons JF, Simpson JW, Srinivasan M, Tartaro KR, Tomasz A, Vogt KA, Volkmer GA, Wang SH, Wang Y, Weiner MP, Yu P, Begley RF, Rothberg JM (2005). Genome sequencing in microfabricated high-density picolitre reactors. Nature.

[71] Weber AP, Weber KL, Carr K, Wilkerson C, Ohlrogge JB (2007). Sampling the Arabidopsis transcriptome with massively parallel pyrosequencing. Plant Physiol.

[72] Torres TT, Metta M, Ottenwalder B, Schlotterer C (2008). Gene expression profiling by massively parallel sequencing. Genome Res.

[73] Troyanskaya OG (2005). Putting microarrays in a context: integrated analysis of diverse biological data. Brief Bioinform.

[74] Joyce AR, Palsson BO (2006). The model organism as a system: integrating 'omics' data sets. Nat. Rev. Mol. Cell Biol.

[75] De Keersmaecker SC, Thijs IM, Vanderleyden J, Marchal K (2006). Integration of omics data: how well does it work for bacteria?. Mol. Microbiol.

